# Salt Intake Is Associated with Inflammation in Chronic Heart Failure

**Published:** 2014-09-01

**Authors:** Alper Azak, Bulent Huddam, Namik Gonen, Seref Rahmi Yilmaz, Gulay Kocak, Murat Duranay

**Affiliations:** 1 Department of Nephrology, Ankara Education and Research Hospital, Ankara, Turkey; 2 Department of Internal Medicine, Ankara Education and Research Hospital, Ankara, Turkey; 3 Department of Nephrology, Hacettepe University Faculty of Medicine, Ankara, Turkey

**Keywords:** Heart Failure, Inflammation, Sodium Dietary

## Abstract

**Background:**

Chronic Heart Failure (CHF) is highly prevalent and is associated with high morbidity and mortality rates. It has been well established that excessive intake of sodium chloride (salt) induced hypertension in some populations. Although salt seems to induce cardiovascular diseases through elevation of blood pressure, it has also been indicated that salt can induce cardiovascular diseases independently from blood pressure elevation.

**Objectives:**

The present study aimed to evaluate the association between salt consumption and inflammation in CHF patients.

**Patients and Methods:**

This study was conducted on 86 patients between 18 and 65 years old who were diagnosed with New York Heart Association (NYHA) functional class I and II heart failure. Salt intake was calculated by using 24 hour urine sodium excretion. Besides, the association between inflammation and daily salt intake was evaluated regarding C - reactive protein (CPR), High sensitive CRP (HsCPR), Erythrocyte Sedimentation Rate (ESR), and ferritin and fibrinogen levels using Pearson correlation analysis.

**Results:**

Our results showed a statistically significant difference between the low (n = 41) and high (n = 45) salt intake groups in terms of serum HsCRP levels (5.21 ± 2.62 vs. 6.36 ± 2.64) (P < 0.048). Additionally, a significant correlation was observed between the amount of salt consumption and HsCRP levels. In this study, daily salt consumption of the enrolled patients was 8.53 gram/day. The medications and even the blood pressures were similar in the two groups, but daily pill count, prevalence of hypertension, and coronary heart disease were higher in the high salt intake group; however, the differences were not statistically significant (P = 0.065). Also, no significant difference was observed between the groups concerning the inflammation markers, such as CRP, ESR, ferritin, and fibrinogen.

**Conclusions:**

Neurohumoral and inflammatory factors are thought to contribute to high mortality and morbidity rates in CHF. Yet, inflammatory markers may early diagnose CHF and predict the prognosis. Excessive salt intake also worsens the inflammation as well as volume control.

## 1. Background

Chronic Heart Failure (CHF) is highly prevalent in the general population and is associated with high morbidity and mortality rates. The prevalence of CHF has been reported to be about 1 - 2% in the general population (1). It has long been recognized that CHF is associated with inflammatory cell activation (2-4).

It has been well established that excessive intake of sodium chloride (salt) induced hypertension in some populations. Although salt seems to induce cardiovascular diseases through the elevation of blood pressure, it has also been known that salt can induce cardiovascular diseases independently from blood pressure elevation (5).

Nutritional factors in heart failure may be related to the pathophysiology of the disease, including inflammation. However, the association between salt consumption and inflammation has not been evaluated in the patients with CHF. Being a major contributing factor to high mortality rates in CHF, inflammation may be enhanced by high salt intake.

## 2. Objectives

Therefore, the present study aims to evaluate the relationship between salt intake and inflammation in the patients with CHF.

## 3. Patients and Methods

This study was conducted on 95 patients between 18 and 65 years old who were diagnosed with New York Heart Association (NYHA) functional class I and II heart failure (6). Nine patients were excluded due to loss of data. The exclusion criteria of the study were the presence of chronic inflammatory disease, infection, uric acid metabolism disorders, malignancy, anemia, diuretic use, and estimated glomerular filtration rate lower than 60 mL/min calculated using the MDRD Formula (7).

Echocardiography was performed before blood samples were collected for serum analysis. Salt intake was computed using 24 hour urinary sodium extraction. Serum levels of High sensitive C - reactive protein (HsCRP), C - reactive protein (CRP), fibrinogen, ferritin, Brain Natriuretic Peptide (BNP), aldosterone, and homocysteine were measured, as well.

### 3.1. Statistical Analysis

The Statistical Package for the Social Sciences (SPSS) 12.0 (SPSS inc., Chicago, IL, USA) was used for all the statistical analyses. The data are presented as mean ± standard deviation for continuous variables, and as percentages for the categorical ones. The scale data between the groups were analyzed using unpaired Student’s T test. In addition, Pearson correlation coefficient was used to analyze the correlations. Besides, one-way ANOVA was used to make comparisons between sodium/creatinin groups.

## 4. Results

This study was conducted on 86 patients (29 males, 57 females). The patients' demographic characteristics, comorbidities, and medications have been listed in Table 1.

**Table 1.** Demographic Characteristics and the Medications of the Patients[Table-fn fn11861]

Sex	Female (n = 45)	Male (n = 41)	Total (n = 86)
**Mean age (years)**	64.51 ± 12.17	65.59 ± 8.02	64.87 ± 10.91
**Diabetes mellitus**	24 (53.3)	10 (24.3)	34
**Hypertension**	40 (88.9)	29 (70.7)	69
**Coronary artery disease**	14 (31.1)	7 (17)	21
**Chronic kidney disease**	4 (8.8)	4 (9.7)	8
**Hyperlipidemia**	13(28.8)	3 (7.3)	16
**ACEI**	14 (31.1)	7 (17)	21
**ARB**	20 (44.4)	5 (12.1)	25
**CCB**	21 (46.6)	10 (24.3)	31
**Beta Blocker**	9 (20)	5 (12.1)	14
**OAD**	15 (33.3)	7 (17)	22
**Insulin**	7 (15.5)	2 (4.8)	9
**ASA**	9 (20)	6 (14.6)	15
**Statin**	9 (20)	3 (7.31)	12

Abbreviations: ACEI, Angiotensin converting enzyme inhibitor; ARB, Angiotensin receptor blocker; CCB, Calcium channel blocker; OAD, Oral anti diabetic; ASA, Acetyl salicylic acid

The patients were divided into three groups according to their 24 hour urine sodium/creatinine ratio (Group I < 100 mEq/g, n = 18, 20.9%; group II 100 - 200 mEq/g, n = 45, 52.3%; group III > 200 mEq/g, n = 23, 26.7%). The 24 hour urine sodium/creatinine ratios were 76.05 ± 12.83, 143.07 ± 28.03, and 252.93 ± 43.49 in groups I, II, and III, respectively. Besides, serum HSCRP levels were higher in group III compared to groups I and II and the difference was statistically significant (Table 2).

**Table 2.** Study Groups' Characteristics Based on Their Urinary Sodium/Creatinine Ratio[Table-fn fn11862][Table-fn fn11863][Table-fn fn11864]

	Group I (< 100 mEq/g, n=27)	Group II (100-200 mEq/g, n= 31)	Group III (>200 mEq/g, n=29)	P value
**Sex (M/F)**	14 / 13	16 / 14	15 / 14	0.086
**Age**	67.44 ± Kas.81	63.56 ± 10.9	65.43 ± 10.29	0.420
**Systolic blood pressure**	141.4 ± Kas.48	145.2 ± 15.2	144.13 ± 17.75	0.673
**Diastolic blood pressure**	83.11 ± 10.57	83.84 ± 6.4	85.65 ± 8.7	0.560
**Antihypertensive agent count/day**	2.3 ± 0.4	2.9 ± 0.5	3.01 ± 0.4	0.460
**CRP (mg/dL)**	0.97 ± 1.03	1.23 ± 1.06	1.97 ± 0.65 ^b^	0.003
**24 hour urine protein (mg/day)**	226.9 ± 579.05	608 ± 218.29	554.2 ± 112.7	0.497
**24 hour sodium/creatinin ratio**	76.05 ± 12.8	143.1 ± 28.03	538.05 ± 197.8^ a, b^	< 0.01
**proBNP (pg/mL)**	109.4 ± 77.2	104.8 ± 70.1	106.97 ± 80.54	0.975
**Aldosterone (pmol/L)**	170.5 ± 64.36	165.4 ± 66.9	152.3 ± 50.42	0.607
**Homocysteine (μmol/L)**	14.8 ± 6.12	15.44 ± 6.75	15.16 ± 6.78	0.941
**Fibrinogen**	397.6 ± 56.03	408.9 ± 69.01	406.48 ± 70.91	0.830
**Ferritin**	73.24 ± 47.29	94.9 ± 63.6	68.63 ± 48.2	0.140
**HsCRP**	5.46 ± 3.36	5.6 ± 3.05	5.14±3.26	0.780
**Erytrocyte sedimantation rate/hour**	22.28 ± 14.7	24.18 ± 17.5	20.3 ± 14.2	0.64
**Hemoglobin**	13.97 ± 1.29	13.85 ± 1.55	13.77 ± 1.32	0.900
**Creatinine**	1.09 ± 0.28	1.13 ± 0.35	0.86 ± 0.15	0.060
**Sodium**	140.6 ± 3.05	140.62 ± 2.8	140.96 ± 2.63	0.870
**Potassium**	4.11 ± 0.048	4.59 ± 0.51	4.55 ± 0.45	0.078
**Uric Acid**	5.54 ± 1.55	5.8 ± 1.77	7.81 ± 0.86^ b, c^	< 0.01
**Albumin**	4.2 ± 0.21	4.14 ± 0.33	4.07 ± 0.4	0.430
**Microalbuminuric patient count**	4	5	4	

^a,^ P < 0.05 Group I vs Group II

^b,^ P < 0.05 Group I vs Group III

^c,^ P < 0.05 Group II vs Group III

**Figure 1.**
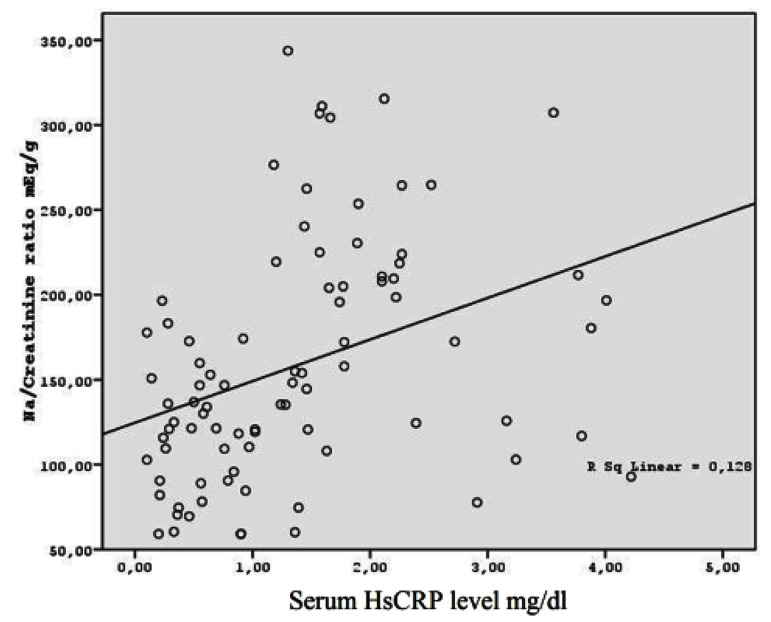
The Correlation between Serum HsCRP Levels and Na/Creatinin Ratio

In order to evaluate the association between inflammation and daily salt intake, the patients were compared in terms of CRP, HsCRP, ESR, ferritin, and fibrinogen. The results indicated a significant difference among the study groups regarding urinary sodium/creatinine ratio and serum HSCRP levels (Figure 1).

Serum uric acid levels were measured as 5.54 ± 1.55, 5.8 ± 1.77, and 7.81 ± 0.86 in groups I, II, and III, respectively. Additionally, higher serum uric acid levels were detected in group III and the difference was statistically significant.

## 5. Discussion

The findings of the present study showed that 24 hour urinary sodium/creatinine ratio was highly correlated to serum HSCRP levels. Also, high sodium intake was associated with higher serum uric acid levels.

In the last decade, some improvements were made in understanding the pathophysiology and treatment of CHF. High mortality and morbidity rates in these patients imply that unexplained mechanisms may also play a role. It has been well known that compensatory mechanisms of CHF involve renal, neurohumoral, and renin-angiotensinaldosterone system. Also, it has been hypothesized that persisted immune system activation and inflammation may have crucial importance. Recent studies have focused on the negative effects of inflammation and circulating cytokines on cardiac inotropy and remodeling in course of CHF. In an animal model of dilated cardiomyopathy, it was shown that circulating inflammatory cytokines were increased (8-10). In SOLVD trial, it was found that CHF patients whose plasma TNF-alpha levels were below 6.5 pg/mL had better survival rates (11). Also, TNF-alpha and IL-6 have been mentioned as independent predictors of mortality in CHF (3, 12).

Studies have also been conducted on the effectiveness of treatments. In PRAISE trial, amlodipine lowered IL-6 but not TNF-alpha and high dose enalapril lowered IL-6 levels. Additionally, ß adrenergic stimulation increased IL-1 and TNF-alpha levels, while ß blockade lowered these inflammatory cytokines. Moreover, statin therapy lowered CRP levels (13).

In general, neurohumoral activation, aldosterone synthesis, and triggers of these two take place in pathophysiological fundamentals of CHF. Aldosterone that is significantly influenced by salt intake plays a role in pathophysiological mechanisms of both CHF and hypertension. Our study results showed no significant difference among the study groups regarding serum aldosterone levels. Also, no statistically significant difference was observed among the groups in terms of demographic characteristics and co-morbidities. 

Dietary salt intake affects vascular endothelial independently from blood pressure. A study demonstrated that excessive salt intake in normotensive rats resulted in higher expression of endothelial TGF-beta (14).

In National Health and Nutrition Examination Survey (NHANES III), the patients with non-ischemic CHF were evaluated in terms of CRP and fibrinogen levels. According to the results, fibrinogen levels were higher in the CHF patients and also displayed a racial difference. In addition, the highest levels of fibrinogen were detected in non-Hispanic whites (15).

Microalbuminuria is a useful prognostic marker in evaluation of both diabetic and non-diabetic patients for renal and cardiovascular risk profiling (15-18). Microalbuminuria is thought to result from vascular endothelial damage and increased vascular permeability in kidneys and, consequently, may be an early predictor of atherosclerosis (19). A large number of studies have confirmed that microalbuminuria may predict cardiovascular mortality (20, 21). Moreover, the studies involving hypertensive and diabetic patients have demonstrated that microalbuminuria is associated with male sex, glycemic control, blood pressure, triglyceride levels, central obesity, smoking, diabetes vintage, and age (22, 23). Nevertheless, the current study indicated no relationships between microalbuminuria and sodium/creatinine ratio. Also, no significant relationship was found between microalbuminuria and salt intake in our study. This insignificant finding might be due to the fact that few patients were involved, even the patients' comorbidities were similar, and the vintage and stages of the diseases were not included. Since microalbuminuria is a strong predictive and prognostic factor for heart diseases, the relationship between this marker and salt intake is thought to be worth evaluating.

BNP is a protein which consists of 32 amino acids. It is primarily secreted as preproBNP after volume and pressure increases in the ventricles and is converted enzymatically into N-terminal proBNP (NT-proBNP) and BNP. Both NT-proBNP and BNP are used for diagnosis of congestive heart failure (24, 25). BNP can also be used as a prognostic marker in acute coronary syndrome (26). It has been shown that BNP may predict sudden cardiac death (25). Nonetheless, the current study results did not indicate any relationship between salt intake and BNP levels. It is possible that not only salt intake but also some other factors may have a role in secretion of BNP.

HsCRP is produced from the liver in case of systemic inflammation and is assumed as a new risk factor for atherosclerosis. Coronary atherosclerosis is manifested by CHF. Alonso-Martinez et al. studied the relationship between NYHA CHF stages and Left Ventricle Ejection Fraction (LVEF) in hospitalized CHF patients. HsCRP levels were found to be reversely correlated to functional capacity and LVEF. Also, higher HsCRP levels were found to predict mortality and rehospitalization (14).

In National Health and Nutrition Examination Survey (NHANES III), fibrinogen and HsCRP levels were found to be higher in the patients with non-ischemic CHF compared to those without CHF (16). Howic et al. demonstrated that statin therapy improved left ventricle performance in ischemic and non-ischemic serious CHF patients. Moreover, HsCRP levels were found to be correlated to higher mortality and rehospitalization rates. In addition to the positive pleiotropic effects of statins, lower HsCRP levels may also be a contributing factor (27).

Our study results showed that salt intake was correlated to HsCRP levels. This implies that increased salt intake may play a role in the neurohumoral and the physiopathological pathways of CHF besides increasing the volume load. Thus, it may be hypothesized that salt restriction may improve the inflammatory process besides improvements in the volume load and hypertension.

Recent studies have supported the effectiveness of inflammatory markers and cytokines in CHF physiopathology by negative inotropic impact and remodeling.

The present study also revealed no relationship between salt intake and other inflammation markers, except for HsCRP. The positive correlation between salt intake and HsCRP makes us think that salt intake may contribute to inflammatory damage in CHF. Yet, the effect of salt restriction on the inflammation status needs to be further investigated.

### 5.1. Limitations

The main limitation of the present study was the absence of a control group. Another limitation of the study was the number of the patients involved in this study. Also, evaluation of salt intake and inflammation by using the stages of CHF was not performed; early and late stages of CHF may show a different association in terms of salt intake and inflammation.
